# Circulating miRNA as Biomarkers for Colorectal Cancer Diagnosis and Liver Metastasis

**DOI:** 10.3390/diagnostics11020341

**Published:** 2021-02-19

**Authors:** Farah J. Nassar, Zahraa S. Msheik, Maha M. Itani, Remie El Helou, Ruba Hadla, Firas Kreidieh, Rachelle Bejjany, Deborah Mukherji, Ali Shamseddine, Rihab R. Nasr, Sally N. Temraz

**Affiliations:** 1 Department of Internal Medicine, Faculty of Medicine, American University of Beirut, Beirut 1107 2020, Lebanon; fjn00@mail.aub.edu (F.J.N.); rla06@mail.aub.edu (R.E.H.); rah117@mail.aub.edu (R.H.); fk30@aub.edu.lb (F.K.); rb62@aub.edu.lb (R.B.); dm25@aub.edu.lb (D.M.); as04@aub.edu.lb (A.S.); 2 Department of Anatomy, Cell Biology and Physiological Sciences, Faculty of Medicine, American University of Beirut, Beirut 1107 2020, Lebanon; zsm10@mail.aub.edu (Z.S.M.); mmi40@mail.aub.edu (M.M.I.)

**Keywords:** colorectal cancer, microRNA, biomarker, diagnosis, liver metastasis

## Abstract

Colorectal cancer (CRC) is the second leading cause of cancer deaths worldwide. Stage IV CRC patients have poor prognosis with a five-year survival rate of 14%. Liver metastasis is the main cause of mortality in CRC patients. Since current screening tests have several drawbacks, effective stable non-invasive biomarkers such as microRNA (miRNA) are needed. We aim to investigate the expression of miRNA (miR-21, miR-19a, miR-23a, miR-29a, miR-145, miR-203, miR-155, miR-210, miR-31, and miR-345) in the plasma of 62 Lebanese Stage IV CRC patients and 44 healthy subjects using RT-qPCR, as well as to evaluate their potential for diagnosis of advanced CRC and its liver metastasis using the Receiver Operating Characteristics (ROC) curve. miR-21, miR-145, miR-203, miR-155, miR-210, miR-31, and miR-345 were significantly upregulated in the plasma of surgery naïve CRC patients when compared to healthy individuals. We identified two panels of miRNA that could be used for diagnosis of Stage IV CRC (miR-21 and miR-210) with an area under the curve (AUC) of 0.731 and diagnostic accuracy of 69% and liver metastasis (miR-210 and miR-203) with an AUC = 0.833 and diagnostic accuracy of 72%. Panels of specific circulating miRNA, which require further validation, could be potential non-invasive diagnostic biomarkers for CRC and liver metastasis.

## 1. Introduction

Colorectal cancer (CRC) is the third most common cancer (10.2%) and the second leading cause of cancer death (9.2%) in both sexes worldwide, representing a major health burden [1]. In Lebanon, CRC ranked as the fifth most common cancer accounting for 8% of the newly diagnosed cancer cases over a period of 11 years (2005–2015) [2]. Patients diagnosed with localized and regional CRC (early stage CRC) have a good prognosis with a five-year survival rate reaching 90%. However, patients with metastatic CRC (Stage IV CRC) have a five-year survival rate of 14% [3]. Although current screening tests help reduce CRC incidence and mortality, they are characterized by several drawbacks including low sensitivity and specificity, high cost, and invasiveness, which can reduce the patient’s compliance [3]. Moreover, liver metastasis is the main cause of mortality in CRC patients [4]. The liver is often the first site of metastatic disease and about 15–25% of new CRC patients will have liver metastases at the initial time of diagnosis. Although the 5-year overall survival rates after hepatectomy for colorectal cancer metastasis is improved (47–60% surviving at 5 years), recurrence occurs in 40–75% of patients after liver resection [5]. As such, it would be essential to find more effective, cheap, and noninvasive biomarkers for the early detection of CRC and continuous monitoring of CRC liver metastasis.

During the past two decades, research has shed light on microRNA (miRNA) as potential players in the development of several human diseases including cancer. miRNA are small, endogenous, noncoding RNA molecules, approximately 18–25 nucleotides in length, that regulate gene expression at the post-transcriptional level by binding to the 3′-untranslated region (3′-UTR) of mRNAs leading to their translational repression or degradation [6]. Extensive research showed that miRNA are consistently altered in CRC tissues compared to normal ones and they play a pivotal role in its initiation, development, and progression [7]. Notably, miRNA expression is also dysregulated in biological fluids including plasma, serum, saliva, and urine taken from cancer patients compared to those from healthy controls. These circulating molecules are characterized to be stable, reproducible, and consistent among individuals of the same species suggesting that they have the potential to serve as noninvasive diagnostic, prognostic, and therapy predictive biomarkers for several malignancies in general [8] and for CRC in particular [9,10,11].

Multiple studies have reported the dysregulation of circulating miR-21, miR-19a, miR-23a, miR-29a, miR-145, miR-203, miR-155, miR-210, miR-31, and miR-345 in CRC patients [12,13,14,15,16,17,18,19,20]. However, most of these studies investigated the miRNA individually on early stage CRC or using heterogeneous CRC population. Therefore, the aim of this study is to determine the expression of these miRNA in the plasma of Lebanese Stage IV CRC patients and to identify panels that could be used for diagnosis of Stage IV CRC in general and liver metastasis in particular.

## 2. Results

### 2.1. Characteristics of Study Subjects

A total of 106 participants were recruited; including 62 Stage IV CRC patients (median age 58 ± 13.49 years) and 44 healthy subjects (median age 41.72 ± 8.97 years) were recruited in the study (Table 1). Out of the 62 patients with Stage IV CRC, 33 CRC patients gave their blood sample at diagnosis before any bowel resection surgery (surgery naïve), while 15 patients gave their blood sample after a recent bowel resection surgery (postoperative). Fourteen of the patients were previously diagnosed with CRC at an early stage and had undergone bowel resection surgery and were recently diagnosed with Stage IV CRC (recurrent). The majority (61.3%) of the CRC patients were males, while 40.9% of the controls were males. All of the healthy subjects (100%) were Lebanese as well as most of the CRC patients (82%). Around 43% of CRC patients had a family history of cancer and 75.8% of the CRC patients had liver metastasis. Tumor location was mainly left-sided (60%) with right-sided (25%) and rectal (15%). As for the most common mutations in CRC patients, 51.9% had KRAS mutations, 6.1% had NRAS mutations, and none had BRAF mutation.

### 2.2. Circulating miRNA Expression in Surgery Naïve Stage IV CRC Patients Compared to Healthy Subjects

**Figure 1:**
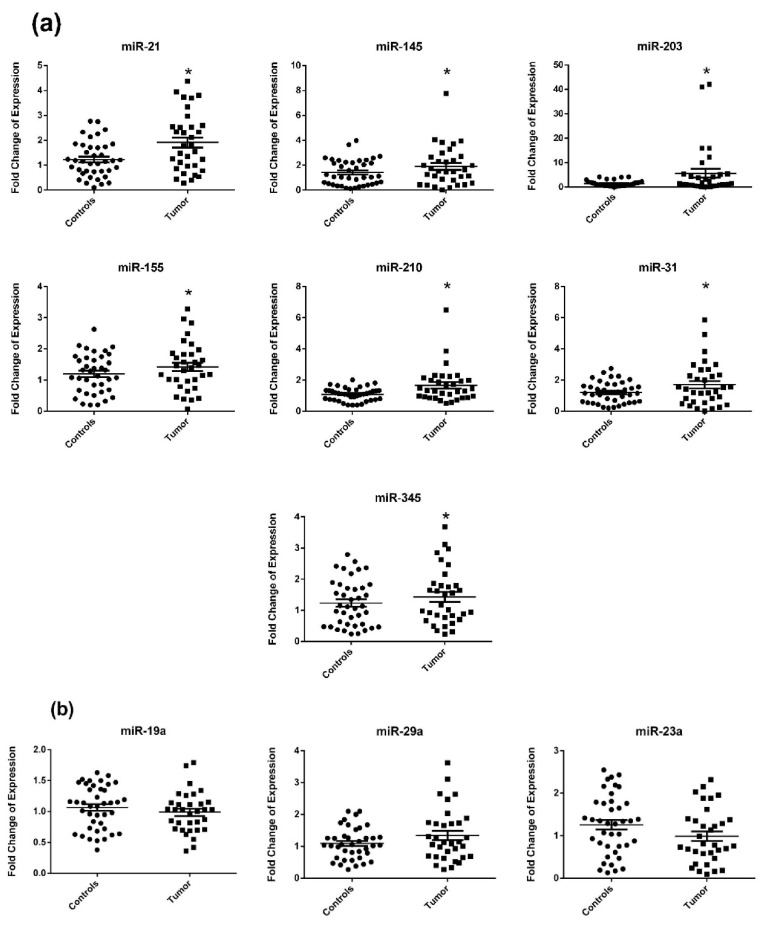
miRNA expression in plasma of newly diagnosed surgery naïve Stage IV colorectal cancer (CRC) patients compared to healthy subjects. (**a**) Significantly dysregulated miRNA (**b**) Non-significantly dysregulated miRNA. The dotplots represent the mean (middle line) and the standard error of mean (error bars). miR-16 was used as an endogenous control. * denotes *p*-value < 0.05 according to Wilcoxon’s signed-rank test.

miRNA expression was measured in the plasma of 33 CRC patients (newly diagnosed and before they had any bowel resection surgery) and compared to their expression in the plasma of 44 healthy subjects. miR-21, miR-145, miR-203, miR-155, miR-210, miR-31, and miR-345 were significantly upregulated in the plasma of surgery naïve CRC patients compared to healthy subjects with a *p*-value of 0.0003, 0.0029, 0.0103, 0.0059, 0.0005, 0.0135, and 0.0319 respectively (Figure 1a). On the other hand, miR-19a, miR-29a, and miR-23a were non-significantly dysregulated in the plasma of surgery naïve CRC patients (Figure 1b).

### 2.3. Circulating miRNA Expression in Surgery Naïve vs. Postoperative and Recurrent Stage IV CRC Patients

**Figure 2:**
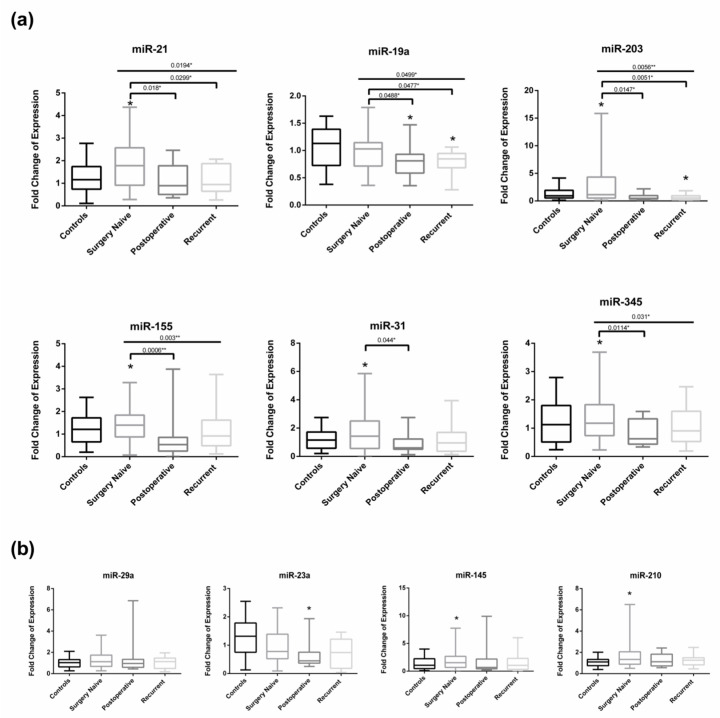
miRNA expression in plasma of surgery naïve Stage IV CRC patients compared to those recently postoperative and those recurrent (meaning newly diagnosed Stage IV CRC previously undergone bowel resection surgery at early stage CRC). (**a**) Significantly dysregulated miRNA (**b**) Non-significantly dysregulated miRNA. Whiskers represent minimum and maximum, the top, the bottom, and the band in the box represent the first and third quartile and the median respectively. * above boxplots denotes *p*-value < 0.05 according to Wilcoxon’s signed-rank test. * above line denotes *p*-value < 0.05 and ** above line denotes <0.01 according to Kruskal Wallis test when comparing all subgroups and to Mann–Whitney test when comparing 2 subgroups.

In order to study the effect of bowel surgical resection, we compared miRNA expression in three groups of Stage IV CRC patients whose plasma was taken at diagnosis (surgery naïve *N* = 33), postoperatively following bowel resection surgery (*N* = 15), or from a recurrent group (*N* = 14) who had already done a bowel resection procedure when diagnosed with early stage CRC but were now newly diagnosed with Stage IV disease. When comparing miRNA expression in plasma of surgery naïve and postoperative CRC patients, miR-21, miR-19a, miR-203, miR-155, miR-31, and miR-345 were significantly upregulated in surgery naïve CRC patients compared to the postoperative ones with respective *p*-value of 0.018, 0.0488, 0.01147, 0.0006, 0.044, and 0.0114 (Figure 2a). Moreover, miR-21, miR-19a, and miR-203 were significantly upregulated in plasma of surgery naïve CRC patients compared to recurrent patients with respective *p*-value of 0.0299, 0.0477 and 0.0051. Figure 2b shows the nonsignificant miRNA dysregulation between the different subgroups. Interestingly, miR-19a was significantly downregulated in postoperative and recurrent CRC patients compared to healthy controls (*p*-value of 0.012 and 0.0012 respectively) and miR-203 was significantly downregulated in recurrent CRC patients compared to healthy controls with *p*-value of 0.0295 (Figure 2a). Moreover, miR-23a was significantly downregulated in postoperative CRC patients compared to healthy subjects with a *p*-value of 0.0099 (Figure 2b).

### 2.4. Diagnostic Accuracy of miRNA for CRC Detection

**Figure 3:**
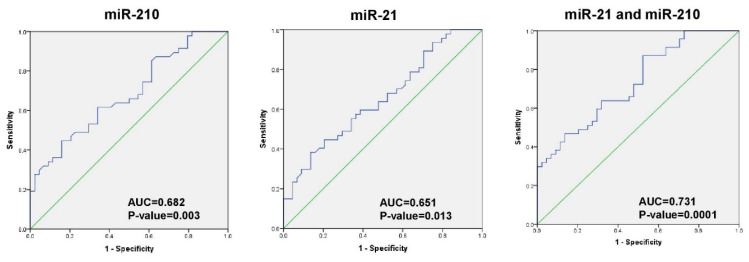
Receiver Operating Characteristics (ROC) curve of miR-210, miR-21 and their combination to differentiate CRC patients from healthy subjects.

In order to evaluate the ability of significantly dysregulated plasma miRNAs to differentiate CRC patients (surgery naïve and recurrent) from healthy subjects, a Receiver Operating Characteristics (ROC) curve was plotted for miRNA with significant AUC area under the curve (AUC) (Figure 3) and the optimal cut-off values, specificity, sensitivity, positive predicted value (PPV), negative predicted value (NPV), and diagnostic accuracy for best diagnostic miRNA were calculated (Table 2). A combined plasma miRNA panel of miR-21 and miR-210 had an AUC of 0.731 ± 0.052 (*p*-value of 0.0001 and 95% CI: 0.63–0.832) and the sensitivity, specificity, and diagnostic accuracy were 87.2%, 47.7%, and 69%, respectively.

### 2.5. Diagnostic Accuracy of miRNA for Liver Metastasis Detection

**Figure 4:**
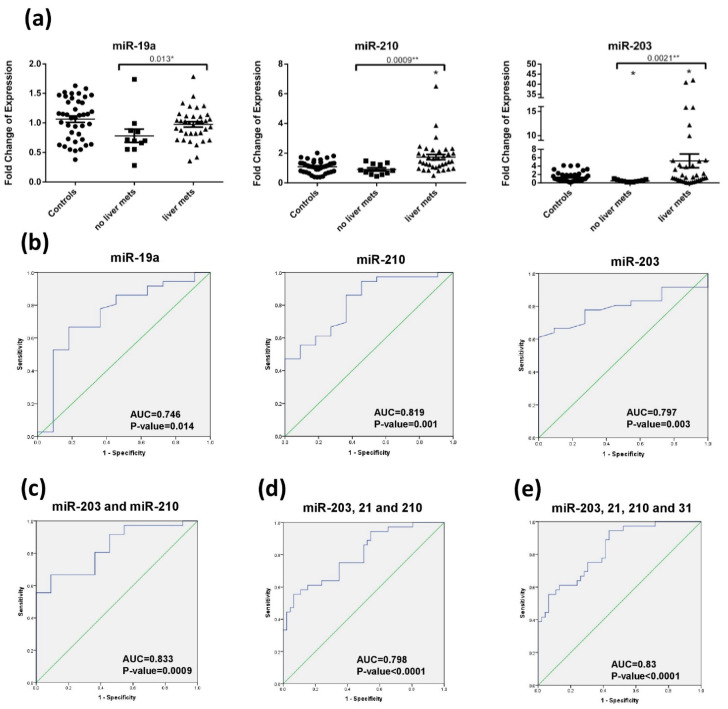
Circulating miRNA for liver metastasis in CRC patients. (**a**) miR-19a, miR-210, miR-203 expression in CRC patients with liver metastasis vs. metastasis to other sites. The dotplots represent the mean (middle line) and the standard error of mean (error bars). * denotes *p*-value < 0.05 according to Wilcoxon’s signed-rank test. * above line denotes *p*-value < 0.05 and ** above line denotes <0.01 according to Mann–Whitney test. mets: metastasis (**b**) ROC curve of miR-19a, miR-210, and miR-203 to differentiate CRC patients with liver metastasis from other metastasis. (**c**) ROC curve of combination miR-210 and miR-203 to differentiate CRC patients with liver metastasis from other metastasis. (**d**) ROC curve of combination miR-203, miR-21, and miR-210 to differentiate CRC patients with liver metastasis to healthy subjects and those CRC with no liver metastasis. (**e**) ROC curve of combination of miR-203, miR-21, miR-210, and miR-31 to differentiate CRC patients with liver metastasis to healthy subjects and those CRC with no liver metastasis.

None of the miRNA were differentially expressed upon dividing the newly diagnosed Stage IV CRC patients (surgery naïve and recurrent) into subgroups according to age groups (below and above 50 years), body mass index (underweight, normal, overweight, obese), smoking habits, alcohol intake, family history of cancer, tumor sidedness, and KRAS mutation (Data not shown). However, when dividing the CRC patients (surgery naïve and recurrent) into those with and without liver metastasis, miR-19a, miR-210, and miR-203 were significantly dysregulated with respective *p*-values of 0.014, 0.001, and 0.003 (Figure 4a). A combination of plasma miR-210 and miR-203 can distinguish between CRC patients with or without liver metastasis with an AUC of 0.833 ± 0.063 (*p*-value: 0.003 with 95% CI: 0.622–0.775) (Figure 4b,c) and a sensitivity, specificity, and diagnostic accuracy of 66.7%, 90.9% and 72%, respectively (Table 3). Notably, as reported in Section 2.2, miR-203 was significantly downregulated in recurrent compared to surgery naïve, while miR-210 was not, so miR-210 alone could be better to differentiate between liver metastasis patients vs. no liver metastasis in the recurrent patients alone.

Upon analyzing whether the studied miRNA could be used to discriminate CRC patients with liver metastasis from healthy subjects and CRC patients with no liver metastasis, miR-210, miR-21, and miR-203 were significantly dysregulated in liver metastatic CRC patients with respective *p*-value of 0.000079, 0.033, and 0.021. A panel of plasma miR-210, miR-21, and miR-203 had a sensitivity, specificity, and diagnostic accuracy of 55.6%, 93.9%, and 71% with an AUC of 0.798 to identify CRC patients with liver metastasis (Figure 4d). miR-31 had a near borderline significant *p*-value (0.057) when comparing CRC patients with liver metastasis with healthy subjects and CRC patients with no liver metastasis. When adding miR-31 to the panel, it gave higher sensitivity 94.4%, lower specificity of 56.5% and a diagnostic accuracy of 73% with an AUC of 0.83 (Figure 4e).

## 3. Discussion

Several studies have been exploring circulating miRNA as potential biomarkers for CRC diagnosis, prognosis and therapy prediction. However, most of the diagnostic studies were concerned with finding these biomarkers for early stage CRC detection [9]. In this study, we investigated the expression of extensively studied circulating miRNAs in Lebanese Stage IV CRC patients and evaluated their potential to be used as diagnostic biomarkers for advanced CRC and its liver metastasis. Our results showed that miR-21, miR-145, miR-203, miR-155, miR-210, miR-31, and miR-345 were significantly upregulated in the plasma of surgery naïve CRC patients when compared to healthy individuals. However, miR-19a, miR-29a, and miR-23a were non-significantly dysregulated in the plasma of the surgery naïve CRC patients. Moreover, we identified three panels of miRNA that could be used for diagnosis of Stage IV CRC (miR-21 and miR-210) and liver metastasis in CRC {(miR-210 and miR-203) and (miR-21, 203, 210 and 31).

miR-21 has been shown to be the most upregulated miRNA in hematological and solid tumors including CRC and it acts as an oncomiR [21]. It was reported to promote cell growth and invasion in CRC by targeting PTEN and PDCD4 [22,23]. In CRC, and consistent with our results, miR-21 is upregulated in tissues and in circulation (plasma and serum) compared to healthy controls [24,25,26]. A recent meta-analysis conducted on 18 studies including 1129 blood specimens of CRC patients and 951 control specimens has reported that circulating miR-21 has a potential diagnostic value with moderate sensitivity 77% and good specificity 83% for CRC [27].

miR-210, a hypoxia-regulated miRNA exhibits oncogenic properties in most cancers, where it mediates cell proliferation, migration, invasion, and clonogenicity [28]. It has been identified along with eight other miRNA as potentially useful diagnostic biomarkers in CRC tissues [29]. Many reported its potential diagnostic value as it is upregulated in the serum of CRC patients similarly to our data in plasma [16,26,30].

As for miR-203, its role and expression in CRC tissues is controversial [31,32]. It acts as a tumor suppressor in CRC that inhibits proliferation, migration, and invasion [33] and plays a role in metastasis acting as a signaling molecule in exosomes between tumors and monocytes in metastatic CRC patients [34]. miR-203 was significantly upregulated in plasma of our Stage IV CRC cases similarly to what is reported in plasma and serum of previous studies [35,36]. Its expression is even higher in Stage III-IV CRC when compared to Stage I-II CRC [35]. A meta-analysis evaluated the diagnostic value of miR-203 for the diagnosis of CRC by pooling the data from nine studies and reported 83% sensitivity and 80% specificity [37]. However, miR-203 was found to be downregulated in serum of CRC patients (majority of Stage II and III) compared to controls and was considered with five other serum miRNAs (miR-21, let-7g, miR-31, miR-92, and miR-181b) to distinguish CRC from controls [38].

miR-31 acts as a promoter of cell proliferation, invasion, and migration in vitro and tumorigenesis and metastasis in CRC [39]. It is reported to be overexpressed in CRC tissues compared to normal tissues [40]. Similar to our results in plasma, it is also upregulated in serum of CRC patients [41] where it exhibits along with miR-146a-5p, and miR-148a-3p strong diagnostic ability [42]. High levels of miR-31 were found in plasma of CRC patients with lymph node metastasis compared to patients without lymph node metastasis or healthy individuals [43]. However, serum miR-31 was reported to be downregulated in another study with a majority of Stage II and III CRC patients [38].

Moreover, we found miR-145, miR-155 and miR-345 to be significantly upregulated in the plasma of surgery naïve CRC patients. miR-145 has been reported to be downregulated in CRC tissues and to act as a tumor suppressor that suppresses proliferation, metastasis, and EMT in CRC [44,45]. The upregulation of miR-145 detected in our samples is not in accordance with the literature. Several studies reported its downregulation in the plasma/serum of CRC patients of different ethnicities when compared to healthy controls and shed its association with clinical stage [14,36,46,47,48,49]. miR-155 is reported to be upregulated in colon cancer tissues compared to normal ones and to be involved in the invasion of colorectal cancer SW-480 cells by regulating the Wnt/β-catenin pathway [18,50]. Our data has shown that miR-155 was significantly upregulated in plasma of surgery naïve Stage IV CRC patients compared to control. This is in accordance with Lv et al. who reported its upregulation in the serum of CRC patients particularly highest in Stage IV when compared to matched healthy controls and its ability to distinguish between these two groups [18]. However, another study showed that miR-155 was downregulated in the serum of patients with colonic and rectal cancer when compared to controls, while being higher in rectal than in colonic cancer [51]. On the other hand, miR-345 has not been studied for CRC diagnosis. It has been reported to be downregulated in CRC tissues with a potential role in suppressing cell proliferation and invasion [52]. High circulating serum miR-345 was found to correlated with unfavorable preoperative chemoradiotherapy response and poor locoregional control in locally advanced rectal cancer [20] and its high expression in blood in metastatic CRC was associated with worse outcome in non-KRAS mutant patients treated with 3rd Line Cetuximab and Irinotecan [53]. However, in our study, it was upregulated in the plasma of surgery naïve Stage IV CRC. As for the other potential oncomiRs in CRC (miR-19a [54], miR-29a [55], and miR-23a [56]) we obtained nonsignificant dysregulation in plasma of Stage IV CRC patients which also contrasts their upregulation in CRC blood/plasma/serum reported in the literature [12,13,24,46,57,58].

Our data showed that miR-19a, miR-210, and miR-203 could distinguish between CRC patients with liver metastasis and those with other metastasis. Increase in exosomal miR-19a in the serum of CRC patients was associated with liver metastasis compared with the low expression group [59]. miR-19a was reported to have a role in invasion and metastasis in vitro by inhibiting Transglutaminase-2 (TG2), a critical cross-linking enzyme in the extracellular matrix (ECM) and tumor microenvironment [60,61]. miR-203 levels have been shown to be upregulated in liver metastasis compared to matched primary CRC tissues. In addition, high serum miR-203 expression was significantly associated with liver metastasis and other metastasis. Serum miR-203 expression significantly discriminated CRC patients with liver metastasis with an AUC = 0.719, sensitivity (61.54%), and specificity (84.18%) in cohort of 24 healthy controls and 186 preoperative CRC samples and an AUC = 0.690, sensitivity (91.67%), and specificity (46.97%) in the validation cohort composed of 144 preoperative CRC samples [15]. Another study reported that high miR-203 expression group correlated with higher incidence of distant metastasis including liver metastasis (*p* < 0.01) as compared to the low expression group. In addition, overexpressing miR-203 in a xenograft mice model promoted more liver metastasis [34]. As for miR-210, microarray analysis identified its upregulation in liver metastasis tissues samples compared to primary CRC tumor tissues [62]. This was also validated by another study that used real time PCR and found out the overexpression of miR-210 in liver metastases compared to primary tumors is associated with lower survival [63]. Recent studies revealed that serum miR-210 levels has diagnostic value to discriminate patients with metastatic tumors to patients with primary hepatocellular carcinoma [64] and in predicting the recurrence and prognosis of CRC hepatic metastasis [65]. Overall, these results show that miR-19a, miR-210, and miR-203 might play a promising role as non-invasive liver metastasis-prognostic biomarkers in patients with metastatic CRC. Interestingly, high levels of miR-21 and miR-31 are present in tissue samples of liver metastasis [66,67]. miR-21 in plasma derived exosomes are positively correlated with liver metastasis in CRC patients [68,69]. Notably, serum miR-21 was identified as CRC liver metastasis-associated miRNA when evaluated in 116 consecutive localized, 72 synchronous liver-metastatic CRC and 36 other organ-metastatic CRC by real-time PCR [70]. These two miRNAs were identified as a panel with miR-210, miR-203 for detection of liver metastasis (in CRC) compared to no liver metastasis (in CRC and healthy).

Some limitations to the use of miRNAs as biomarkers lie in the fact that CRC itself is a heterogeneous disease and the expression of miRNAs vary significantly within tumor population. Also, the lack of consensus on suitable small RNA reference genes to quantify circulating miRNA levels makes establishment of global cut-off value difficult. Finally, some miRNAs are highly expressed in the blood cells, and levels of circulating miRNAs can be significantly altered by hemolysis. Thus, the standardization of protocols and methodologies, including sample storage, RNA extraction, and quantification of circulating miRNAs, are necessary to improve the biomarker potential of miRNAs.

## 4. Materials and Methods

### 4.1. Specimen Collection

After Institutional Review Board (IRB) approval of the study (BIO-2018-0041), sixty-two CRC patients and 44 healthy controls were recruited from the American University of Beirut Medical Center between May 2018 and September 2020. Written informed consent was obtained from all participants. CRC patients were all newly diagnosed Stage IV that were confirmed by biopsy or radiological imaging. None of the patients has a history of autoimmune or inflammatory bowel diseases and none had undergone chemotherapy before sample collection. The healthy controls had no history of cancer or autoimmune and inflammatory bowel diseases. All patient clinicopathological characteristics were collected from their medical records. Whole blood was collected from each participant into EDTA tube for miRNA analysis. Plasma was subsequently isolated by double centrifugation steps and then stored at −80 °C until further step.

### 4.2. Total RNA Extraction

Total RNA was extracted from 250 μL plasma using Plasma/Serum Circulating and Exosomal RNA Purification Kit (Norgen Biotek Corp., Thorold, ON, Canada) according to the manufacturer’s instructions. Assessment of RNA concentration and quality was done using DeNovix DS-11 FX spectrophotometer (Wilmington, DE, USA). The total RNA samples were stored at −80 °C until analysis.

### 4.3. miRNA Expression Using Reverse Transcription Quantitative Real Time Polymerase Chain Reaction (RT-qPCR)

For reverse transcription and RT-qPCR, validated primers and probes from TaqMan^®^ microRNA Assays Kit (Applied Biosystems, Waltham, MA, USA) were used for hsa-miR-16, hsa-miR-21, hsa-miR-19a, hsa-miR-29a, hsa-miR-23a, hsa-miR-145, hsa-miR-203, hsa-miR-155, hsa-miR-210, hsa-miR-31, and hsa-miR-345. cDNA was synthesized for the miRNA of interest starting from 10 ng of extracted RNA using TaqMan^®^ MicroRNA Reverse Transcription Kit (Applied Biosystems, Waltham, MA, USA) according to the manufacturer’s instructions. RT-qPCR was then carried out in duplicates for each sample using 2x TaqMan^®^ Universal Master Mix with no Amperase Uracil N-glycosylase (UNG) (Applied Biosystems, Waltham, MA, USA) as previously described [71]. RT-qPCR was performed using BioRad CFX96 Real Time System, C1000 Thermal Cycler (Hercules, CA, USA). The cycling conditions were 10 min at 95 °C then 40 cycles of: 15 s at 95 °C and 60 s at 60 °C. The fold change of miRNA expression was calculated using the ∆∆Ct equation where miR-16 is endogenous control and compared to healthy controls.

### 4.4. Statistical Analysis

All statistical analyses were performed using SPSS version 22 (Armonk, NY, USA) and GraphPad Prism 6 software (San Diego, CA, USA). Baseline characteristics were presented as mean ± standard deviation (SD) for continuous data and as number (percentages) for categorical parameters. Wilcoxon signed rank test was used as a non-parametric one sample test when comparing the fold change of expression of the miRNA to that of healthy subject considered as 1. Mann-Whitney U and Kruskal-Wallis tests were applied to detect any significant dysregulation of the miRNA within two or more clinicopathological subgroups. Diagnostic accuracy of candidate miRNAs or their combinations was assessed by Receiver Operating Characteristic (ROC) curves analysis using ∆Ct values or predicted probability calculated from binary logistic regression analysis. In order to identify the best cut-off, sensitivity and specificity for each miRNA, Youden’s index was calculated. *p*-values less than 0.05 were considered statistically significant.

## 5. Conclusions

In conclusion, this is the first study to report circulating miRNA expression in Lebanese Stage IV CRC patients with identification of miRNA panels that could distinguish surgery naïve CRC patients from healthy controls and that could differentiate between CRC with liver metastasis and those with other metastasis or healthy controls. Taken together, our data suggests that circulating miRNA could be potential non-invasive diagnostic biomarkers for CRC and liver metastasis. Further validations from larger, independent cohorts are needed and comparability with other routine tests are required.

## Figures and Tables

**Table 1:** Distribution of Clinicopathological Data for Stage IV CRC patients and healthy subjects. Data were presented as N (%) or as Mean ± SD. BMI: Body Mass Index, Hx: history; mets: metastasis.

Variable	All CRC Patients	Surgery Naïve	Postoperative	Recurrent	Healthy Subjects
Sample	62 (58.5)	33 (53.2)	15 (24.2)	14 (22.6)	44 (41.5)
*Age* (years)	58 ± 13.49	56.3 ± 2.2	56.26 ± 13.86	63.9 ± 14.25	41.72 ± 8.97
*BMI (*kg/m^2^*)*	27.17 ± 6.34	27.6 ± 6.37	25.1 ± 7.66	28.37 ± 4.35	26.39 ± 0.39
*Gender*					
Male	38 (61.3)	21 (63.6)	6 (40)	11 (78.6)	18 (40.9)
Female	24 (38.7)	12 (36.4)	9 (60)	3 (21.4)	26 (59.1)
*Nationality*					
Lebanese	50 (82)	27 (81.8)	12 (85.7)	11 (78.6)	44 (100)
Iraqi	8 (13.1)	5 (15.2)	1 (7.1)	2 (14.3)	
Other	3 (4.9)	1 (3)	1 (7.1)	1 (7.1)	
*Smoking habits*					
No	47 (75.8)	26 (78.8)	11 (73.3)	11 (78.6)	27 (61.4)
Yes	15 (24.2)	7 (21.2)	4 (26.7)	3 (21.4)	17 (38.6)
*Alcohol intake*					
No	48 (77.4)	26 (78.8)	11 (73.3)	11 (78.6)	34 (81)
Yes	14 (22.6)	7 (21.2)	4 (26.7)	3 (21.4)	8 (19)
*Family Hx of Cancer*					
No	35 (57.4)	19 (57.6)	7 (46.7)	9 (64.3)	
Yes	26 (42.6)	14 (42.4)	8 (53.3)	4 (30.8)	
*Tumor Sidedness*					
Left	36 (60)	24 (72.7)	6 (42.9)	6 (42.9)	
Right	15 (25)	3 (9.1)	7 (50)	5 (35.7)	
Rectum	9 (15)	5 (15.2)	1 (7.1)	3 (21.4)	
*Liver Mets*					
No	15 (24.2)	5 (15.2)	4 (26.7)	6 (42.9)	
Yes	47 (75.8)	28 (84.8)	11 (73.3)	8 (57.1)	
*KRAS mutation*					
No	26 (48.1)	16 (53.3)	5 (33.3)	6 (50)	
Yes	28 (51.9)	14 (46.7)	8 (53.3)	6 (50)	
*NRAS mutation*					
No	46 (93.9)	28 (93.3)	9 (100)	10 (90.9)	
Yes	3 (6.1)	2 (6.7)	0	1 (9.1)	
*BRAF mutation*					
No	49 (100)	28 (100)	9 (100)	11 (100)	
Yes		0	0	0	

**Table 2:** Diagnostic parameters to evaluate individual and combined miRNA for diagnosis of CRC patients. AUC: area under the curve, SE: standard error of mean, PPV: positive predicted value, NPV: negative predicted value, and DA: diagnostic accuracy.

miRNA	AUC	SE	*p*-Value	95% CI	Youden’s Index	Cut-Off	Sensitivity (%)	Specificity (%)	PPV (%)	NPV (%)	DA (%)
miR-210	0.682	0.055	0.003	0.574–0.79	0.288	10.46	44.7	84.1	75	56	62
miR-21	0.651	0.057	0.013	0.539–0.76	0.247	2.81	38.3	86.4	75	53	59
miR-210+21	0.731	0.052	0.0001	0.63–0.832	0.35	0.464	87.2	47.7	65	79	69

**Table 3:** Diagnostic parameters to evaluate individual and combined miRNA for liver metastasis in CRC patients. AUC: area under the curve, SE: standard error of mean, PPV: positive predicted value, NPV: negative predicted value, and DA: diagnostic accuracy.

miRNA	AUC	SE	*p*-Value	95% CI	Youden’s Index	Cut-Off	Sensitivity (%)	Specificity (%)	PPV (%)	NPV (%)	DA (%)
*miR-19a*	0.746	0.091	0.014	0.569–0.924	0.485	6.025	66.7	81.8	93	45	72
*miR-210*	0.819	0.069	0.001	0.684–0.955	0.497	11.115	86.1	63.6	89	58	81
*miR-203*	0.797	0.063	0.003	0.673–0.92	0.611	15.65	61.1	100	100	44	70
*miR-210+203*	0.833	0.063	0.0009	0.711–0.956	0.576	0.838	66.7	90.9	96	45	72

## Data Availability

The data presented in this study are available on request from the corresponding author. The data are not publicly available due to patient data privacy and ongoing research.
